# Atopic diseases in children and adolescents are associated with behavioural difficulties

**DOI:** 10.1186/s12887-021-02663-7

**Published:** 2021-04-23

**Authors:** Wiebke Keller, Mandy Vogel, Freerk Prenzel, Jon Genuneit, Anne Jurkutat, Cornelia Hilbert, Andreas Hiemisch, Wieland Kiess, Tanja Poulain

**Affiliations:** 1 Department of Women and Children’s Health, Hospital for Children and Adolescents and Center for Pediatric Research (CPL), Leipzig University, Liebigstrasse 20a, 04103 Leipzig, Germany; 2 LIFE Leipzig Research Center for Civilization Diseases, Leipzig University, Philipp-Rosenthal-Strasse 27, 04103 Leipzig, Germany; 3 Pediatric Epidemiology, Department of Pediatrics, Medical Faculty, Leipzig University, Liebigstrasse 20a, 04103 Leipzig, Germany

**Keywords:** Allergy, Behavioural difficulties, Children, Adolescents

## Abstract

**Background:**

Atopic diseases and behavioural difficulties in children have both been on the rise in recent decades. This study seeks to assess associations between atopic diseases and behavioural difficulties, examining the differences considering child age and how behavioural difficulties were reported (via self-report or parent-report).

**Methods:**

Data on behavioural difficulties, assessed through the Strengths and Difficulties Questionnaire (SDQ), and on atopic diseases, assessed through the participant’s medical history, were available for 2701 study participants aged 3 to 18 years. Associations between atopic diseases and behavioural difficulties were evaluated using linear regression analyses. We split the study sample into two groups. I: 3-to 10-year-olds/parent-reported SDQ (*n* = 1764), II: 11- to 18-year-olds/parent-reported SDQ (*n* = 937) and self-reported SDQ (*n* = 915). All analyses were adjusted for age, gender, and socioeconomic status.

**Results:**

In younger children, atopic dermatitis was strongly associated with higher total difficulties scores, more emotional problems and conduct problems, and more symptoms of hyperactivity/inattention. Parents reported higher total difficulties scores, more emotional problems, and more peer-relationship problems for adolescents with bronchial asthma and other allergies, whereas the adolescents themselves reported more peer relationship problems.

**Conclusion:**

In younger children, atopic dermatitis is associated with internalizing and externalizing problems. In adolescents, bronchial asthma and other allergies are associated with a greater level of internalizing problems only. The findings further suggest that parents of adolescents are more likely to perceive associations between atopic diseases and behavioural difficulties than the adolescents themselves.

## Background

Atopic diseases are common among children and adolescents [1], with both bronchial asthma (BA) and atopic dermatitis (AD) becoming increasingly prevalent in recent years [2–4]. In Germany, the prevalence of BA is approximately 6.3% and the prevalence of AD approximately 14.3% [4]. Each of these diseases can cause a range of problems, including physical, psychological and social impairment [5]. Like atopic diseases, behavioural difficulties are already common and are becoming increasingly prevalent, with 17.2% of children and adolescents in Germany currently affected [6]. Both atopic diseases, especially BA and AD, and behavioural difficulties usually develop during childhood and adolescence [7, 8].

Generally, children and adolescents with chronic illness are at a higher risk of developing mental health problems such as depression and anxiety [9]. As a common form of chronic impairment in childhood and adolescence, atopic disease is no exception [10]. Previous research postulated a higher likelihood for behavioural difficulties in children and adolescents with atopic diseases due to social implications [11], negatively affected brain development [12, 13] or side effects of allergy medication [14].

Regarding AD, several different studies reported that children and adolescents with AD have a higher prevalence of behavioural difficulties compared to healthy children and adolescents, for example conduct problems, emotional problems and increased symptoms of hyperactivity/inattention [5, 15, 16]. Although most studies postulate that AD precedes the development of behavioural difficulties, it is likely that the relationship is bidirectional [17, 18]. The relationship between AD and attention-deficit/hyperactivity disorder (ADHD) has been of particular interest and many studies have found a strong association between these conditions [19, 20].

Concerning BA, a recently published German study found that people with BA have a higher risk of suffering from anxiety, social phobias and affective disorders [3]. Furthermore, an association has been reported between BA and ADHD [19]. Moreover, Goodwin et al. (2013) proposed that the risk of developing behavioural difficulties is correlated with the severity of BA [21]. Therefore, it is possible that the association of BA and behavioural difficulties is not significant for children and adolescents with merely mild BA [21]. At this point, it must be mentioned that the most recent report of GINA – the Global Initiative for Asthma – recommends categorizing patients in epidemiological studies or clinical trials by the type of treatment that they are prescribed [22]. Goodwin et al. (2013), however, assessed the severity of asthma by parental report of clinical symptoms [21], possibly leading to differing results concerning asthma severeness.

Goodwin et al. (2013) further demonstrates that the relationship between BA and behavioural difficulties is only valid for children and adolescents with current BA, indicating that children and adolescents who have suffered from BA in the past but have “outgrown” it over time are not at a higher risk of experiencing behavioural difficulties [21].

Similar results have been reported for other allergies, such as allergic rhinitis, allergic conjunctivitis and food allergies. Like AD, allergic rhinitis and conjunctivitis seem to be associated with ADHD [19], while food allergies in children and adolescents appear to be related to internalizing problems [23], and externalizing difficulties [24].

Although several studies have investigated associations between atopic diseases and behavioural problems, most of these have focused on one specific disease or behavioural difficulty (i.e. AD, ADHD etc). Our study aims to investigate associations between various atopic diseases and several behavioural difficulties. Furthermore, to the best of our knowledge, no previous study has investigated whether associations between atopic diseases and behavioural difficulties differ depending on the informant (child or parent). Previous studies report a possible parent-child disagreement in the reporting of behavioural difficulties [25, 26]. This suggests that associations between atopic diseases and behavioural difficulties might also be influenced by the informant. Therefore, our study investigates how these associations vary depending on whether it is the children and adolescents themselves or their parents who are reporting on behavioural difficulties. For the assessment of behavioural difficulties, we used an instrument (Strengths and Difficulties Questionnaire - SDQ) for which differences in child- and parent - reports have been observed in previous studies [25, 26].

We hypothesized a positive association between atopic diseases and behavioural difficulties, particularly between atopic diseases and both internalizing and hyperactivity/inattention problems. Moreover, we hypothesized that this association might differ depending on the age of children and adolescents. Given that reports on behavioural difficulties of parents versus children were shown to differ significantly, we furthermore hypothesized varying results concerning these associations depending on the informant of behavioural difficulties.

## Methods

### Study sample

**Fig. 1:**
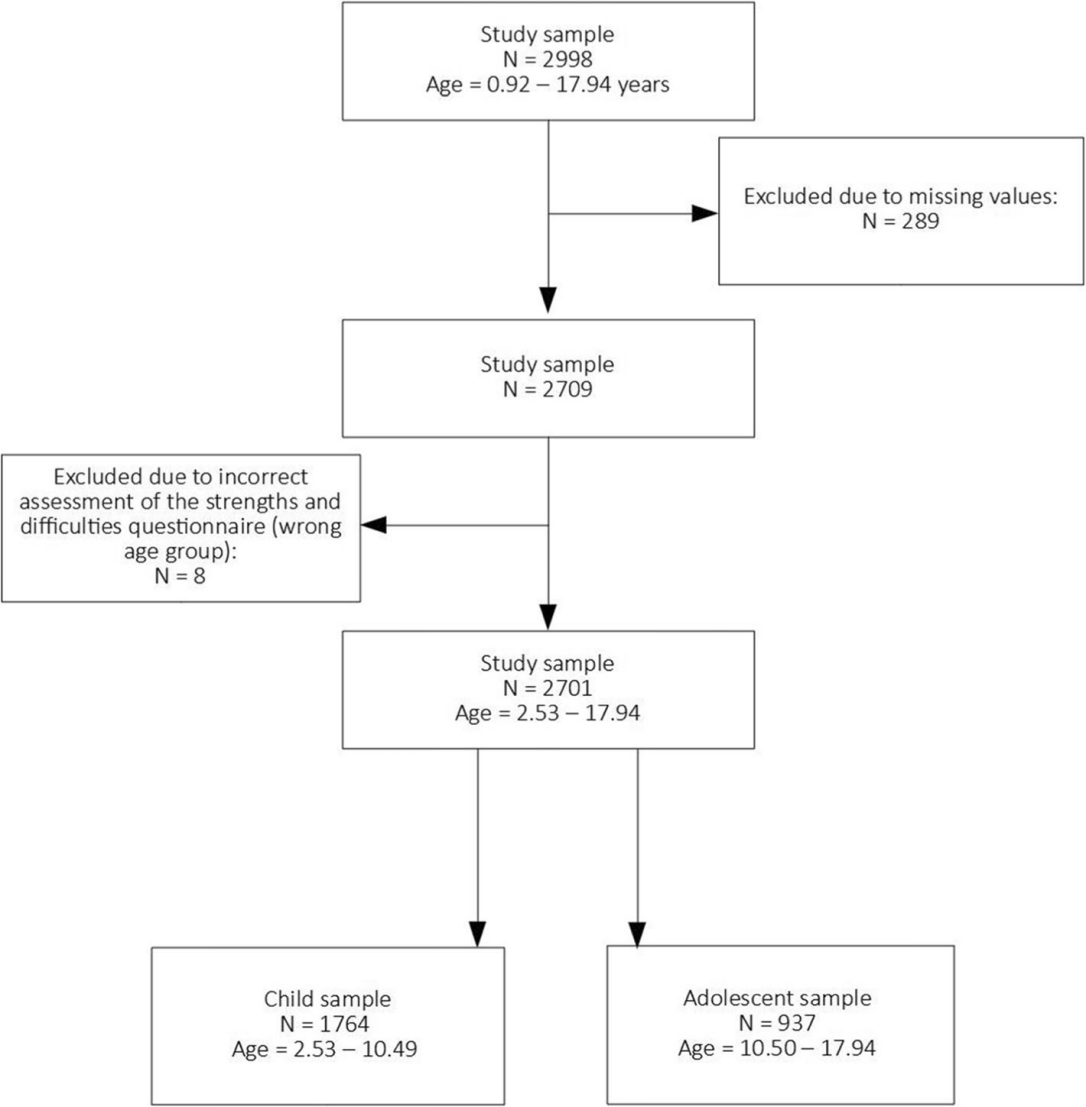
Composition of the study sample in the present study accounting for exclusion criteria

This study is part of the LIFE Child study, a prospective, longitudinal cohort study exploring normal child development, atopic diseases and obesity in children and adolescents [27]. The present project is a cross-sectional study using data collected between 2011 and 2017. We included data for all study participants aged 3 to 18 years who had provided information on atopic diseases and behavioural difficulties. In the case of multiple study visits per child, only the data collected at the first study visit were included. In the case of siblings attending the study, only the youngest child was considered. We split our study sample into two groups. The first group (child sample) included children aged 3 to 10 years (mean age = 6.1, range = 2.5–10.5) with behavioural difficulties assessed by parents. In this group, 1764 children (936 boys and 828 girls) were analysed. The second group (adolescent sample) included 937 adolescents (454 boys and 483 girls) aged 11 to 18 years (mean age = 13.3, range = 10.5–17.9), whose behavioural difficulties were assessed both by themselves (self-report) and by their parents (parent-report). In this age group, self-reported information was missing for 22 adolescents, resulting in a subsample of 915 adolescents (444 boys and 471 girls). For more detailed information concerning the study sample, see Fig. 1.

### Allergies

In order to assess atopic diseases, the study participants’ parents were interviewed about the children’s and adolescent’s medical history and whether they suffered from BA, AD or any other allergies (AOA, i.e. allergic rhinitis, allergic conjunctivitis, food allergies). In doing so, we did not differentiate by the severity of the disease. A child was defined as having an atopic disease if the parents informed that the diagnosis was confirmed by a physician.

### Behavioural difficulties

To assess behavioural difficulties, we used the Strengths and Difficulties Questionnaire (SDQ), as it is a well-established screening instrument for the assessment of behavioural difficulties and exists in a parent-, teacher- and self-report version. The questionnaire contains 25 items in five sub-scales: “emotional problems”, “conduct problems”, “hyperactivity/inattention”, and “peer relationship problems” (representing “difficulties”) as well as the sub-scale “prosocial behaviour” (representing “strengths”) [28]. Scores for the four difficulties sub-scales are added to produce a total difficulties score [28]. Each sub-scale produces a score ranging from 0 to 10 points. Consequently, the total difficulties score ranges from 0 to 40 points. A higher score indicates a greater degree of behavioural difficulties (or strengths) [28]. Another advantage of the SDQ is the categorization into two dimensions, combining the “emotional problems” and “peer relationship problems” sub-scales as an “internalizing” dimension and the “conduct problems” and “hyperactivity/inattention” sub-scales as an “externalizing” dimension [29]. In the present study, only the difficulties scales and the total difficulties score were analysed, as our focus was on children’s and adolescent’s behavioural difficulties rather than strengths. We used the parent-report and self-report versions of the questionnaire.

### Socioeconomic status

Socioeconomic status (SES) was assessed using the Winkler-Stolzenberg-Index, which summarizes information about each parent’s education, occupational status and monthly net household income. Winkler-Stolzenberg-Index scores can be classified into three categories: 3–8.4 points equates to low SES, 8.5–15.4 points to medium SES and 15.5–21 points to high SES [30]. In the case of differing scores between mother and father, the higher score was used in the analysis.

### Statistical analysis

Data analysis was performed using R version 3.4.3 [31]. Associations between behavioural difficulties and atopic diseases were evaluated using multiple linear regression analyses. The single behavioural difficulties scales of the SDQ were included as dependent variables, and either BA, AD or AOA was included as an independent variable. All analyses were controlled for age, gender, and SES.

## Results

### Descriptive analysis

Table 1 shows the percentages of the different atopic diseases and the average behavioural difficulties scores, separated by sample. In the child sample, 5.8% (*n* = 102) suffered from BA, 17.4% (*n* = 307) suffered from AD and 12.0% (*n* = 212) suffered from AOA. In the adolescent sample, 9.1% (*n* = 85) suffered from BA, 26.4% (*n* = 247) suffered from AD and 23.4% (*n* = 219) suffered from AOA. Concerning behavioural difficulties, the highest total difficulties scores were seen in the adolescent sample (self-report) with a mean total difficulties score of 10.8 (SD = 5.1). The lowest scores were seen in the adolescent sample (parent-report) with a mean total difficulties score of 9.0 (SD = 6.0). In all samples, the highest sub-scale scores were observed in the “hyperactivity/inattention” sub-scale (see Table 1). In both the child sample and the adolescent sample (parent-report), the lowest scores were seen in the “peer relationship problems” scale. In contrast, in the adolescent sample (self-report), the lowest scores were observed in the sub-scale “conduct problems” (see Table 1).

**Table 1:** Description of allergies and behavioural difficulties in the present sample

	Child sample^a^ (*n* = 1764)	Adolescent sample^b^ (*n* = 937)	
**Age (mean (SD))**	6.1 (2.5)	13.3 (1.9)	
**Gender (n (%))**	male: 936 (53.1)	male: 454 (48.5)	
	female: 828 (46.9)	female: 483 (51.5)	
**SES**^**c**^			
**Low**^**c**^ **(n (%))**	208 (11.8)	162 (17.3)	
**Medium**^**c**^ **(n(%))**	1031 (58.4)	574 (61.3)	
**High**^**c**^ **(n(%))**	525 (29.8)	201 (21.4)	
		*Parent-report*	*Self-report*
**SDQ total difficulties score (mean (SD))**	9.4 (5.2)	9.0 (6.0)	10.8 (5.1)
**SDQ sub-scale “emotional problems” (mean (SD))**	1.9 (1.9)	2.4 (2.1)	2.7 (2.1)
**SDQ sub-scale “peer relationship problems” (mean (SD))**	1.4 (1.6)	1.8 (1.9)	2.4 (1.8)
**SDQ sub-scale “hyperactivity/ inattention” (mean (SD))**	3.9 (2.4)	3.0 (2.4)	3.8 (2.1)
**SDQ sub-scale “conduct problems” (mean (SD))**	2.1 (1.6)	1.9 (1.7)	1.9 (1.5)
**Bronchial Asthma (n (%))**	102 (5.8)	85 (9.1)	85 (9.3)
**Atopic Dermatitis (n (%))**	307 (17.4)	247 (26.4)	238 (26.0)
**Any other allergies (n (%))**	212 (12.0)	219 (23.4)	217 (23.7)

^a^Child sample = children aged 3 to 10 years (parent-report version of the SDQ)

^b^Adolescent sample = adolescents aged 11 to 18 years (parent-report version and self-report version of the SDQ)

^c^Winkler-Stolzenberg-Index (WSI), low SES = WSI 3–8.4, medium SES = WSI 8.5–15.4, high SES = WSI 15.5–21

### Associations between behavioural difficulties and atopic diseases

**Fig. 2:**
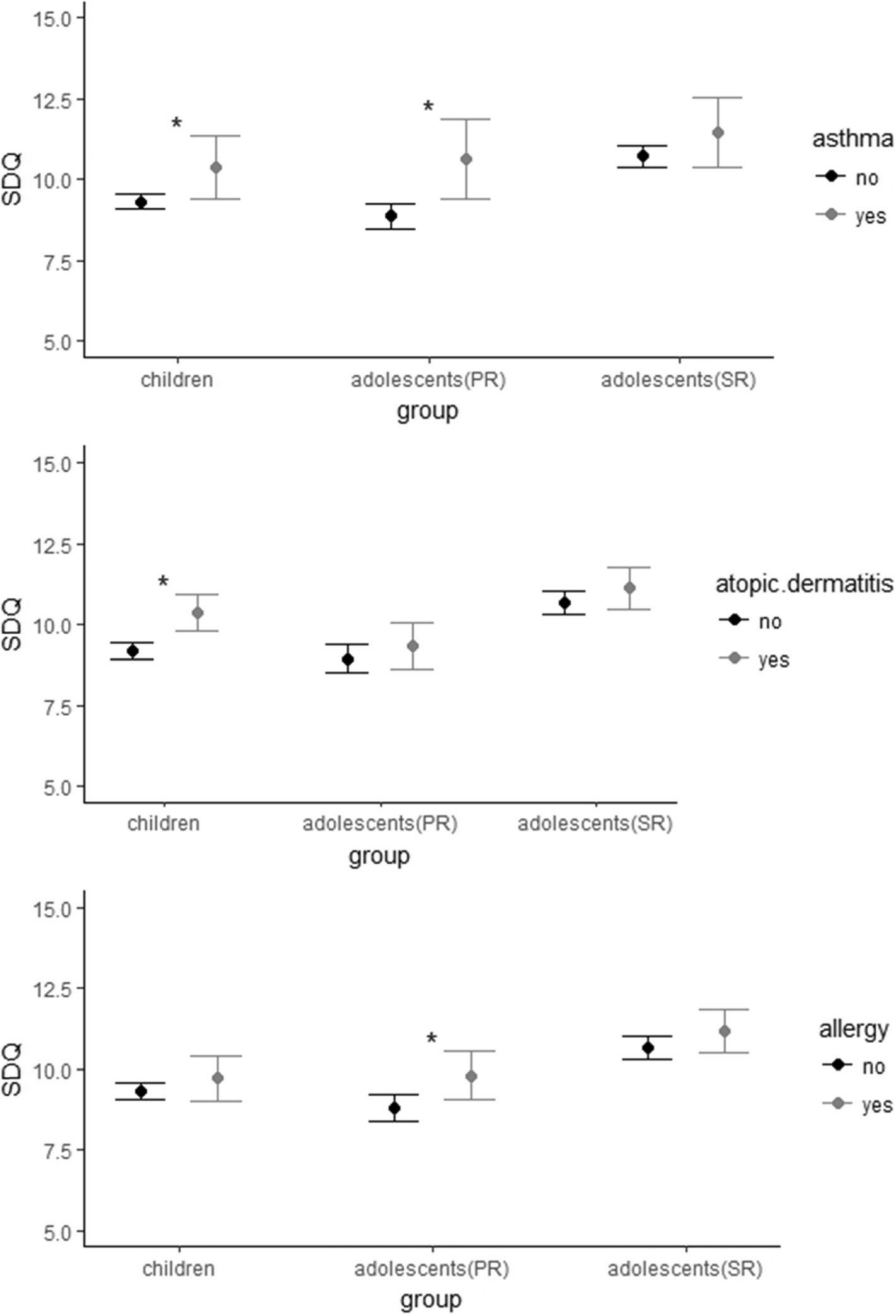
Association between bronchial asthma, atopic dermatitis and any other allergies and SDQ total difficulties scores, adjusted for age, gender, and socioeconomic status

Tables 2, 3 and 4 show the regression coefficients, confidence intervals and *p*-values for the regression analyses. The results are visualized in Fig. 2.

**Table 2:** Associations between bronchial asthma and behavioural difficulties in the different samples

	Child sample^**a**^		Adolescent sample^**b**^ ***Parent - report***		Adolescent sample ***Self-report***	
	b^**c**^ (CI (95%)^**d**^)	***p***	b (CI (95%))	***p***	b (CI (95%))	***p***
**Emotional Problems**	0.39 (0.02–0.75)	0.037	0.55 (0.08–1.02)	0.022	0.24 (− 0.21–0.69)	0.299
**Peer Relationship Problems**	0.19 (− 0.13–0.51)	0.235	0.69 (0.26–1.11)	0.001	0.58 (0.18–0.98)	0.004
**Hyperactivity/ Inattention**	0.28 (− 0.19–0.74)	0.239	0.27 (− 0.24–0.77)	0.299	− 0.02 (− 0.48–0.45)	0.948
**Conduct Problems**	0.21 (− 0.10–0.53)	0.189	0.26 (− 0.12–0.64)	0.178	− 0.05 (− 0.37–0.27)	0.761

All associations are adjusted for age, gender, and socioeconomic status

^a^Child sample = children aged 3 to 10 years (parent-report version of the SDQ)

^b^Adolescent sample = adolescents aged 11 to 18 years (parent-report and self-report version of the SDQ)

^c^b = regression coefficient, non-standardized

^d^*CI* confidence interval

**Table 3:** Associations between atopic dermatitis and behavioural difficulties in the different samples

	Child sample^**a**^		Adolescent sample^**b**^ ***Parent - report***		Adolescent sample ***Self-report***	
	b^**c**^ (CI (95%)^**d**^)	***p***	b (CI (95%))	***p***	b (CI (95%))	***p***
**Emotional Problems**	0.42 (0.19–0.65)	< 0.001	− 0.08 (− 0.38–0.23)	0.623	0.02 (− 0.27–0.32)	0.874
**Peer Relationship Problems**	0.19 (− 0.01–0.39)	0.063	0.23 (− 0.04–0.51)	0.095	0.12 (− 0.14–0.39)	0.359
**Hyperactivity/ Inattention**	0.36 (0.07–0.65)	0.016	0.13 (− 0.20–0.46)	0.442	0.13(− 0.17–0.44)	0.393
**Conduct Problems**	0.24 (0.04–0.44)	0.018	0.12 (− 0.12–0.37)	0.321	0.16 (− 0.05–0.37)	0.133

All associations are adjusted for age, gender, and socioeconomic status

^a^Child sample = children aged 3 to 10 years (parent-report version of the SDQ)

^b^Adolescent sample = adolescents aged 11 to 18 years (parent-report and self-report version of the SDQ)

^c^b = regression coefficient, non-standardized

^d^*CI* confidence interval

**Table 4:** Associations between any other allergies and behavioural difficulties in the different samples

	Child sample^**a**^		Adolescent sample^**b**^ ***Parent - report***		Adolescent sample ***Self-report***	
	b^**c**^ (CI (95%)^**d**^)	***p***	b (CI (95%))	***p***	b (CI (95%))	***p***
**Emotional Problems**	0.10 (−0.16–0.37)	0.455	0.28 (− 0.04–0.60)	0.084	− 0.02 (− 0.33–0.29)	0.906
**Peer Relationship Problems**	− 0.01 (− 0.23–0.23)	0.989	0.51 (0.22–0.79)	< 0.001	0.35 (0.08–0.63)	0.01
**Hyperactivity/ Inattention**	0.25 (− 0.09–0.59)	0.143	0.08 (− 0.26–0.42)	0.638	0.09 (− 0.23–0.40)	0.589
**Conduct Problems**	0.03 (− 0.20–0.27)	0.770	0.11 (− 0.14–0.37)	0.378	0.11 (− 0.11–0.33)	0.340

All associations are adjusted for age, gender, and socioeconomic status

^a^Child sample = children aged 3 to 10 years (parent-report version of the SDQ)

^b^Adolescent sample = adolescents aged 11 to 18 years (parent-report and self-report version of the SDQ)

^c^b = regression coefficient, non-standardized

^d^*CI* confidence interval

### Child sample

Both BA and AD were associated with higher scores in the “total difficulties score” (b = 1.07, *p* = 0.038 for BA and b = 1.20, *p* < 0.001 for AD) and the sub-scale “emotional problems” (b = 0.39, *p* = 0.037 for BA and b = 0.42, *p* < 0.001 for AD). Furthermore, AD was associated with higher scores in the sub-scales representing externalizing problems, i.e. “hyperactivity/inattention” (b = 0.36, *p* = 0.016) and “conduct problems” (b = 0.24, *p* = 0.018). The presence of AOA was not correlated with any behavioural difficulties in this group. The greatest effect sizes were found with BA and AD in relation to the “emotional problems” sub-scale.

### Adolescent sample

In the adolescent sample (parent-report), BA was associated with higher parent-reported total difficulties scores (b = 1.76, *p* = 0.007) as well as higher scores in the sub-scales representing internalizing problems, i.e. “emotional problems” (b = 0.55, *p* = 0.022) and “peer relationship problems” (b = 0.69, *p* = 0.001). No significant associations were found with AD. The presence of AOA was associated with higher total difficulties scores (b = 0.34, *p* = 0.026) and higher scores in the sub-scale “peer relationship problems” (b = 0.51, *p* < 0.001). Associations between allergies and the externalizing sub-scales were not significant.

In the adolescent sample (self-report), BA and AOA were associated with higher scores in the sub-scale “peer relationship problems” (b = 0.58, *p* = 0.004 for BA and b = 0.35, *p* = 0.01 for AOA). No other significant associations were found between behavioural difficulties and atopic diseases.

### Comparison of the groups

First, it is interesting to note that AD was only associated with behavioural difficulties in the child sample. In the adolescent sample, we did not observe any associations between AD and behavioural difficulties. Second, in the child sample none of the atopic diseases was associated with a greater level of peer relationship problems, whereas in the adolescent sample, the associations between atopic disease and peer relationship problems were stronger than all other associations. Taking the effect sizes into consideration, it is noticeable that the effect sizes were higher in the adolescent sample (parent-report) than in the child sample, indicating that associations between atopic diseases and behavioural difficulties – as judged by parents – are stronger in older children and adolescents than they are in younger children.

Comparing the parent-report and self-report versions in the adolescent sample, it should be noted that BA and AOA were associated with more peer-relationship problems with both versions. In the sample in which behavioural difficulties were judged by parents, more associations reached significance and effect sizes were higher, indicating that associations between atopic diseases and behavioural difficulties were stronger if child behaviour was judged by parents.

## Discussion

The aim of the present study was to investigate associations between behavioural difficulties and atopic diseases in children and adolescents aged 3–18 years, with a particular focus on the differences between these associations depending on child age and – in the age group of 11- to 18-year-old adolescents – on the version of the questionnaire applied (parent- versus self-report).

### Prevalence of atopic diseases

Compared to the average prevalence of BA (6%) and AD (14%) in children and adolescents aged 0 to 17 years in Germany [4], a slightly higher percentage of children and adolescents in our study suffered from BA (6% in 3- to 10-year-olds and 9% in 11- to 18-year-olds) and AD (17% and 23%, respectively). This might be due to a relatively high percentage of families of high SES in our study (30%), since it has been suggested that high SES is a risk factor for atopic diseases [32]. Furthermore, the study was conducted in a large city with more than 500,000 inhabitants and it has been suggested that living in an urban area might be associated with an increased risk of atopic disease [33, 34].

### Behavioural difficulties

Overall, the distributions of the SDQ scores in the present study were comparable with scores observed in other representative German samples [35, 36], with a small tendency for higher scores in the present study.

### Association between atopic dermatitis and behavioural difficulties

We found that, in children aged 3 to 10 years, AD was associated with a greater level of emotional problems, conduct problems and hyperactivity/inattention problems. This coincides with a study by Hammer-Helmich et al. (2016), who performed a large cross-sectional study on associations between mental health and atopic diseases. Atopic diseases were assessed through a questionnaire by the ISAAC-study and behavioural difficulties were assessed by the SDQ [5]. The authors found that not only AD but also BA was associated with more emotional, conduct and hyperactivity/ inattention problems [5]. It has been reported that the itching caused by AD can lead to chronic sleep disturbance [12], which might increase the risk of neurodevelopmental disorders [13]. Additionally, the itching itself could cause fidgety and agitated behaviour, which might result in (or at least be interpreted as) externalizing behavioural difficulties of the kind described above.

In our study, AD was only associated with behavioural difficulties in this younger age group, not in adolescents. It is known that AD symptoms tend to be more severe in younger children and recede in later childhood [4]. Therefore, young children with AD might be more severely affected by pruritus and itching, which could result in greater behavioural difficulties.

### Association between bronchial asthma and behavioural difficulties

We observed associations between BA and behavioural difficulties – primarily internalizing problems – in both age groups. Although an association between BA and internalizing problems has been described in two previous cross-sectional studies conducted in North America [37, 38], many studies also proposed an association with externalizing problems [19, 39, 40]. Here, no such association was found. However, it must be noted that symptoms of hyperactivity/ inattention were assessed by the SDQ, which is merely a screening tool, not a diagnostic instrument. In contrast, the aforementioned studies (that described associations between bronchial asthma and externalizing problems) mainly included clinical diagnosis of ADHD, which might explain the discrepancy to our results.

A possible underlying mechanism in the relationship between BA and internalizing problems is that young people who suffer from BA may be frustrated because of restricting symptoms such as shortness of breath, wheezing and coughing, which could make depressive or anxious symptoms more likely. Since the association between behavioural difficulties and atopic diseases is probably bidirectional, it should also be mentioned that stress caused by behavioural difficulties and other forms of adversity can lead to asthma attacks [41]. Stress triggering BA has been hypothesized to occur through neurobiological and immunological pathways [42]. Moreover, anxiety and stress increase cortisol and norepinephrine levels, which can lead to an amplified inflammatory response during allergen exposure, leading in turn to increased cortisol release, potentially creating a vicious cycle [43]. Another possible underlying mechanism is that allergy medication, such as oral corticosteroids and inhaled beta-sympathomimetics, leukotriene antagonists and antihistamines, might lead to neuropsychiatric adverse events [14].

### Association between AOA and behavioural difficulties

With the adolescent group, associations between the presence of AOA and peer relationship problems were found for both the parent-report data and self-report data. This agrees with previous research reporting an association between AOA (such as allergic rhinitis, allergic conjunctivitis or food allergies) and greater levels of internalizing problems, for example Nanda et al. (2016). However, unlike our study sample, they only included children at risk of developing allergic diseases (for instance children of allergic parents) that were later assessed by physical examination, skin prick test and/or parental questionnaires [44]. Furthermore, they did not apply the SDQ but the Behaviour Assessment for Children – Second Edition (BASC-2), which differs from the SDQ but also allows the distinction between externalizing and internalizing problems [44]. Comparable to our results, another German study by Genuneit et al. (2014), who conducted a prospective birth cohort study and assessed atopic diseases as well as ADHD by physician’s diagnosis and parental reports, found no relationship between AOA and ADHD as an externalizing problem [17]. A possible reason for associations between AOA and internalizing problems is that chronic atopic diseases decrease the sufferer’s health-related quality of life and are accompanied by various lifestyle limitations and social and financial implications that could lead to internalizing problems [11].

### Parent-report vs. self-report

With both SDQ versions, we found an association between peer relationship problems and BA and AOA respectively. Moreover, in the adolescent sample, we found no association between AD and behavioural difficulties when using either of the SDQ versions. However, a relationship was found between BA and emotional problems when using the parent-report SDQ data that was not present with the self-report SDQ data. Furthermore, the total difficulties scores for adolescents with BA or AOA based on the parent-reports were higher than the scores based on the self-report data. This suggests that parents of young people with BA and/or AOA are more likely to perceive behavioural difficulties in their children than the adolescents are themselves. This theory is strengthened by the stronger effect sizes in the adolescent sample (parent-report) in comparison to the adolescent sample (self-report). In line with our results, Canning et al. (1992) suggests that parents of chronically ill children (in this case diabetes mellitus, cystic fibrosis, inflammatory bowel diseases or cancer) tend to report higher rates of behavioural difficulties in their children than the children and adolescents do themselves [45]. Parents of children and adolescents with atopic diseases may worry more about their child’s disease and its potential dangers, i.e. an asthma attack or anaphylactic shock. It is possible that they transfer their own worries on their child when answering the questionnaires. In comparison, the affected adolescents themselves might be less anxious and more carefree, possibly because they have grown up with, and are used to, the limitations on their physical activity, food consumption et cetera.

Overall, it must be considered that our study results – especially the comparison between parent-report and self-report - are dependent on the questionnaire applied and that they might differ based on the instrument used to assess data.

### Study limitations

All results should be interpreted within the context of study limitations. First, bidirectional effects have been shown or suggested, but our cross-sectional design does not allow us to detect the directionality of effects. Second, we did not include previously described possible mediators in the association of atopic diseases and behavioural difficulties, such as quality and quantity of children’s and adolescent’s sleep, children’s and adolescent’s medication or parent’s mental health problems. Third, study participants from families of low SES were slightly under-represented in this study. Fourth, the data on atopic diseases is based on the parent’s report of a physician’s diagnosis, so there is the possibility of under- or overdiagnosis of atopic diseases producing inaccuracies. Furthermore, data on behavioural difficulties is based on the SDQ, which is a screening questionnaire but no diagnostic instrument. It must also be considered that associations between atopic diseases and behavioural difficulties as well as variations depending on the informant might differ depending on the instrument used to assess data. Finally, it must be noted that the high prevalence of atopic diseases in our study could lead to a bias in the above-mentioned results. The main strengths of our study were the large size of our study sample and the categorization of atopic diseases into three phenotypes (bronchial asthma, atopic dermatitis, any other allergies), allowing us to show differences in their individual association with behavioural difficulties.

## Conclusion

On the one hand, our study confirms the existing data on associations between atopic disorders and behavioural difficulties in children and adolescents. On the other hand, it also shows that this connection cannot be generalized across different age groups and may depend on who is providing the information. We found varying results in different age groups (stronger associations between atopic dermatitis and externalizing disorders in younger children and stronger associations between bronchial asthma or other allergies and internalizing disorders in adolescents). Moreover, the results of our analysis differed depending on the SDQ version applied. We were able to show that the parents of adolescents with bronchial asthma or other allergies tend to perceive a greater level of internalizing problems on the part of their children than the adolescents themselves do.

## Data Availability

The datasets generated and/or analysed during the current study are not publicly available due to ethical restrictions. The LIFE Child study is a study collecting potentially sensitive information. Publishing data sets is not covered by the informed consent provided by the study participants. Furthermore, the data protection concept of LIFE requests that all (external as well as internal) researchers interested in accessing data sign a project agreement. Researchers that are interested in accessing and analysing data collected in the LIFE Child study may contact the data use and access committee (dm@life.uni-leipzig.de).

## Abbreviations

AD: Atopic dermatitis
ADHD: Attention-deficit/hyperactivity disorder
AOA: Any other allergies
BA: Bronchial asthma
SDQ: Strengths and difficulties questionnaire
SES: Socioeconomic status
